# Asian network for molecular diagnosis of primary immunodeficiencies

**Published:** 2011-01-10

**Authors:** YL Lau

**Affiliations:** 1 Department of Paediatrics & Adolescent Medicine, LKS Faculty of Medicine, The University of Hong Kong, Queen Mary Hospital, Hong Kong SAR

## 

Primary immunodeficiency disorders (PIDs) are inborn errors of the immune system. There are over 150 types of PIDs and because of their rarity, multi-center collaboration for pooled data analysis and molecular studies is important to gain meaningful insights about the phenotypic and genetic diversities of PIDs. Since 2001, our unit established collaboration with 30 pediatric centers in China and Southeast Asia to provide e-consultation and free molecular diagnosis for PIDs. It is imperative to organize the data systematically to yield information on epidemiology of PIDs in the region.

By Aug 2010, we have performed genetic tests for 392 patients referred to us and 243 patients (62%) have their genetic mutations identified. 107 patients (27%) were from Hong Kong, 222 (57%) were from mainland China while the rest were from Taiwan, Singapore, Malaysia, Thailand, the Philippines and Australia. In addition, 200 carriers were identified from 331 potential carriers in these 49 families. X-linked agammaglobulinemia (n=90), Wiskott-Aldrich syndrome (n=49), X-linked chronic granulomatous disease (CGD, n=28), X-linked hyperIgM (n=17) and X-linked severe combined immunodeficiency (SCID, n=19) constituted majority of cases. We also identified mutations of rare PIDs, such as autosomal-recessive SCID (IL7R, JAK3, RAG2 and DCLRE1C), autosomal-recessive CGD (NCF1, CYBA), defects of IL12/IFN-gamma axis in patients susceptible to mycobacterial infections, FOXP3 mutation in immunodysregulation- polyendocrinopathy-X-linked (IPEX) syndrome, SH2D1A mutations in X-linked lymphoproliferative syndrome, TACI in common variable immunodeficiency, ITGB2 mutations in leucocyte adhesion deficiency, and AIRE gene mutation in autoimmune polyendocrinopathy with candidiasis and ectodermal dysplasia (APECED). However, mutations could not be identified in about 40% of patients despite distinct clinical and immunological phenotypes. This is the group of patients that would probably yield novel genes responsible for PIDs with appropriate strategic analysis, combining both functional and genomic analysis.

Establishment of PID data and referral network is an initial step to multi-center collaboration. This constitutes the foundation for PID research and documentation of prevalence, disease burden and outcome of patients with PIDs in Asia, as well as identifying new genes critical for immunohomeostasis.

